# A novel taspine derivative suppresses human liver tumor growth and invasion *in vitro* and *in vivo*

**DOI:** 10.3892/ol.2013.1452

**Published:** 2013-07-09

**Authors:** NAN WANG, LEI ZHENG, YINGZHUAN ZHAN, YANMIN ZHANG

**Affiliations:** School of Medicine, Xi'an Jiaotong University, Xi'an, Shaanxi 710061, P.R. China

**Keywords:** tas1611, liver tumor, invasion, matrix metalloproteinases

## Abstract

Taspine is an attractive target of research due to the anticancer and anti-angiogenic effects shown by *in vitro* and *in vivo* experiments. The present study investigated the role of tas1611, which is a derivative of taspine that has increased activity and solubility, in the regulation of the invasive properties of the SMMC-7721 liver cell line *in vitro* and in tumor inhibition *in vivo*. The proliferation of the SMMC-7721 cells was examined using the tetrazole blue colorimetric method. Matrigel^®^ invasion chamber assays and zymogram analyses were performed to assess the inhibitory effect of tas1611 on cell invasion. Finally, a solid tumor athymic mouse model was employed to further investigate the anti-tumor effect of this compound. The results revealed that tas1611 had a marked inhibitory effect on the invasion of the SMMC-7721 cells and that this effect was associated with the activity and expression levels of matrix metalloproteinase (MMP)-2 and MMP-9. Furthermore, tas1611 was able to inhibit tumor growth effectively in a solid tumor SMMC-7721 athymic mouse model. In conclusion, tas1611 may serve as a promising novel therapeutic candidate for the treatment of metastatic liver cancer.

## Introduction

Hepatocellular carcinoma (HCC) is one of the most frequent malignant tumors and its morbidity and mortality rates have risen (1). The majority of cancer-related mortalities, including those that are a result of HCC, are not caused by the growth of the primary tumor, but by the invasive spread of cancer cells to a secondary site (2). Tumor metastasis occurs by a series of steps, including vessel formation, cell attachment, invasion and cell proliferation (3). Tumor cells must move through and degrade the surrounding tissue barriers to escape the primary site and colonize secondary organs. Therefore, the degradation of basement membranes and the extracellular matrix (ECM) is a crucial step in tumor metastasis. This process requires various cellular proteolytic enzymes, among which matrix metalloproteinases (MMPs) are an important family of proteinases that are responsible for the destruction of the ECM. Among the 20 MMPs that have been identified, MMP-2 (gelatinase-A) and MMP-9 (gelatinase-B) are able to efficiently degrade native collagen types IV and V, fibronectin, entactin and elastin. Therefore, the two proteases are considered crucial for cell invasion and the overexpression of MMP-2/−9 is closely associated with a poor prognosis in patients (4–7).

Taspine was screened for the first time from Radix et *Rhizoma leonticis*, termed ‘Hong Mao Qi’ (HMQ) in Chinese, using cell membrane chromatography in the laboratory of the School of Medicine (Xi'an Jiaotong University, Xi'an, Shaanxi, China) and is now known to exhibit a variety of biological properties, including bacteriostasis, antibiosis and antiviral, anti-inflammatory, anti-ulcer and anti-cancer effects (8,9). The anticancer and anti-angiogenic properties of taspine have been demonstrated and the compound may be an ideal candidate for a chemotherapeutic agent (10). Tas1611 is a ring-opened and biphenyl derivative of taspine with increased activity and solubility. The present study analyzed the tumor invasion cascade in the metastatic process and investigated the effect of the tas1611 on the invasion of SMMC-7721 human liver cancer cells and the enzymatic degradation of the extracellular matrices in order to understand the mechanisms of its anti-metastasis effect.

## Materials and methods

### Reagents

Tas1611 was provided by the Natural Drug Research and Engineering Center of Xi'an Jiaotong University (Shaanxi, China). A stock concentration of tas1611 (20 mM) was prepared using dimethyl sulfoxide (DMSO) and stored at 4°C. The stock solution was further diluted with serum-free RPMI-1640 medium immediately prior to being used. 3-(4,5-Dimethylthiazol-2-yl)-2,5-diphenyltetrazolium bromide (MTT) was purchased from Amresco (Cleveland, OH, USA). The RPMI-1640 medium was purchased from Sigma-Aldrich (St. Louis, MO, USA). All the antibodies were purchased from Cell Signaling Technology (Danvers, MA, USA). The Total RNA kit was obtained from Shanghai Fastagen Biotechnology Co., Ltd. (Shanghai, China) and the Revert AID™ first strand cDNA synthesis kit was from Fermentas (Hanover, Lithuania).

### Cells

The SMMC-7721 human liver cell line was purchased from the Shanghai Institute of Cell Biology of the Chinese Academy of Sciences (Shanghai, China). The SMMC-7721 cells were cultured in RPMI-1640 supplemented with 10% FBS and incubated at 37°C in a 5% CO_2_ atmosphere.

### Mice

BALB/c nude mice (4–6 weeks old) were supplied by the Experimental Animal Center of Xi'an Jiaotong University. The mice were housed and cared for under the standard conditions, with a 12-12 h day/night cycle. Laboratory food and water were freely available. All procedures were carried out in accordance with the guidelines on the care and use of laboratory animals set out by the Xi'an Jiaotong University Animal Ethics Committee (Xi'an, China).

### Cell Culture

The SMMC-7721 human liver cancer cell line was cultured in RPMI-1640 medium (Gibco, Invitrogen Corp., Carlsbad, CA, USA) supplemented with 10% (v/v) FBS in a humidified atmosphere of 5% CO_2_ at 37°C.

### Cell viability assay

The effect of tas1611 on the viability of the SMMC-7721 cells was evaluated using the MTT assay (11). Briefly, the exponentially growing cells were harvested and plated in 96-well plates at a concentration of 2×10^4^ cells/well. Following a 24-h incubation period at 37°C, the cells were treated with various concentrations of tas1611 (0, 0.4, 2, 10 and 50 M) for 48 h. Subsequently, 20 μl MTT (5 mg/ml) was added to each well and the cells were incubated at 37°C for 4 h. Once the supernatant was discarded, 150 μl DMSO was added to each well and the optical density of the cells was determined using a microplate reader (BioRad Instruments, Berkeley, CA, USA) at 490 nm and expressed as absorbance values (12).

### Invasion assay

The invasive potential of the SMMC-7721 cells was assessed in 24-well chemotaxis chambers (Millipore, Billerica, MA, USA) that were pre-coated with 100 μl Matrigel^®^ (1 mg/ml; BD Biosciences, Bedford, MA, USA). The cells were suspended in 200 μl serum-free medium, loaded into the upper chamber and allowed to pass through a polyethylene terephthalate filter with 8-μm pores. The lower chamber was filled with complete medium. The cells that failed to pass through the filters were removed by scrubbing with cotton swabs after 24 h (invasion assay). The cells on the undersurface were fixed in methanol and stained with 0.5% Crystal Violet (Beijing Chemical works, Beijing, China), then images were captured and the cells were quantified in 10 random fields per membrane (13).

### Zymogram analysis of MMP activity

Gelatin zymography was performed as described previously (14). In brief, the SMMC-7721 cells were cultured in RPMI-1640 supplemented with 10% FBS until confluency. The cells were washed three times with PBS and incubated in a serum-free medium or a medium that contained the indicated concentration of tas1611 for 24 h. The media were collected and centrifuged for 10 min at 4°C and 356 × g. The supernatant was mixed with sodium dodecyl sulfate (SDS) sample buffer without a reducing agent, incubated at room temperature for 15 min and loaded on 10% acrylamide gels containing 1% gelatin (Sigma). Following the electrophoresis procedure, the gels were washed with 2.5% Triton X-100 to remove the excess SDS and incubated at 37°C for 20 h in 10 mM Tris-HCl (pH 7.5), containing 150 mm NaCl and 5 mM CaCl_2_. The gels were stained with 0.25% (w/v) coomassie blue G-250 and then destained in 20% methanol containing 10% acetic acid. The areas of protease activity were detected as clear bands against the blue gelatin background. The experiments that were performed in the presence of 10 mM EDTA in the incubation buffer resulted in the abolishment of all zones of gelatin digestion, confirming that the enzymes were metalloproteinases. The amount of protein that was loaded onto the gels was within the linearity of the enzymatic activity. The quantification and comparison of the gelatinolytic activity (relative intensity of the lysis bands) of MMP-9 and MMP-2 were performed by densitometry analysis using an image quantitative analysis system (Image-Pro Plus; Media Cybernetics, Rockville, MD, USA).

### Western blot analysis

Subsequent to the cells being lysed, a standard western blot analysis was performed to investigate the abilities of MMP-2 and MMP-9. The SMMC-7721 cell lines that were treated with 0, 3.3 or 10 μM tas1611 for 48 h were extracted using a cell lysis buffer on ice. The protein concentration was determined using the BCA Protein Quantification kit (Joincare Biosciences, Zhuhai, China) according to the manufacturer's instructions. SDS-polyacrylamide gel electrophoresis (PAGE) was performed in 10% tricine gels loading 40 mg cell lysates per lane. Following the electrophoresis procedure, the separated proteins were transferred to nitrocellulose membranes and blocked using 5% skimmed milk in Tris-buffered saline Tween-20 (TBST) buffer for 2 h. The membranes were then incubated with primary antibodies (MMP-2, MMP-9 and GAPDH polyclonal rabbit antibodies; 1:1,000 dilution) in 5% skimmed milk overnight at 4°C with continuous agitation. Subsequently, the membranes were incubated with secondary anti-rabbit antibodies conjugated with horseradish peroxidase (1:20,000 dilution) for 2 h at room temperature according to the manufacturer's instructions. The blots were detected using an enhanced chemiluminescence (ECL) reagent (Amersham Pharmacia Biotechnology, Piscataway, NJ, USA) and analyzed using Quantity One 1D Analysis software (version 4.4; BioRad) (15,16).

### RNA extraction and quantitative PCR

MMP-2 and MMP-9 mRNA expression in the SMMC-7721 cells was evaluated using quantitative PCR with glyceraldehyde 3-phosphate dehydrogenase (GAPDH) as the housekeeping gene. The SMMC-7721 cells were treated with tas1611 (0, 0.2 and 10 μM) for 48 h. The total RNA was isolated using the Total RNA Extraction kit (Shanghai Fastagen Biotechnology Co., Ltd.) and reverse-transcribed in a 20-μl reaction solution using the First Strand cDNA Synthesis kit (Takara, Shiga, Japan). Each reaction was conducted in 96-well plates with a final volume of 20 μl consisting of 10 μl SYBR Green PCR Master Mix (Takara) and 1 μl of each 2-μM primer. The sequences of the individual pairs of primers of MMP-2, MMP-9 and GAPDH are as follows: MMP-2 forward, 5′-CTCATCGCAGATGCCTGGAA-3′ and reverse, 5′-CAGCCTAGCCAGTCGGATTTG-3′; MMP-9 forward, 5′-ACGCACGACGTCTTCCAG-3 and reverse, 5′-CCACCTGGTTCAACTCACTCC-3′; and GAPDH forward, 5′-AAGGCTGTGGGCAAGGTCATC-3′ and reverse, 5′-GCGTCAAAGGTGGAGGAGTGG-3′. Thermal cycling and fluorescence detection were conducted on a Thermal Cycler Dice^®^ Real Time System (Takara), in accordance with the manufacturer's instructions, at 94°C for 2 min, followed by 40 cycles of 94°C for 20 sec, 55°C for 20 sec and 72°C for 40 sec. Each reaction was performed in triplicate (17).

### Anti-tumor effect of tas1611 on SMMC-7721 cell lines xenografted in athymic mice

The SMMC-7721 cells (2×10^7^ cells/ml) were implanted into the right axilla of athymic mice (0.2 ml/mouse) to form a solid tumor. The athymic mice that developed solid tumors were randomly divided into groups and were administered Tas1611 [100 mg/kg and 200 mg/kg in 0.5% sodium carboxymethyl cellulose (CMC-Na); n=8] or vehicle alone (0.5% CMC-Na; n=8). The drugs were administered once a day for two weeks when the tumor volumes were noticeable. The tumors were measured using calipers every three days and the tumor volume was calculated as follows: Tumor volume = (length × width^2^)/2. The weight of the mice and the tumor volume were recorded when the mice were sacrificed. Animal care was in accordance with the institutional guidelines.

### Statistical analysis

All the values are presented as the mean ± standard error of the mean (SEM) and were analyzed for statistical significance using an analysis of variance (ANOVA). The statistics were determined with an ANOVA and P<0.05 was considered to indicate a statistically significant difference.

## Results

### Tas1611 suppresses SMMC-7721 cell growth

**Figure 1:**
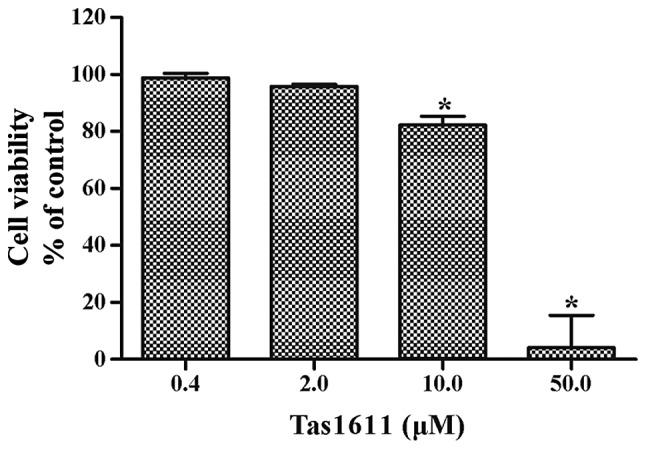
Tas1611 inhibition of SMMC-7721 cell proliferation. The SMMC-7721 cells were treated with or without tas1611 for 48 h. A dose-dependent inhibition of cell number was observed. Data are presented as the mean ± SEM of three separate experiments. ^*^P<0.05 vs. control. SEM, standard error of the mean.

The effects of tas1611 on the viability of the SMMC-7721 cells were examined using an MTT assay. Fig. 1 shows the dose-dependent inhibition of viability by tas1611. The mean cell viability was 98.81±1.56, 95.83±0.69, 82.28±3.06 and 4.20±11.25% at 0.4, 2.0, 10.0 and 50.0 μM, respectively. The 50% growth inhibitory concentration (IC50) of tas1611 on the SMMC-7721 cells was 12.03 M.

### Tas1611 inhibits the invasive properties of the of SMMC-7721 cells

**Figure 2:**
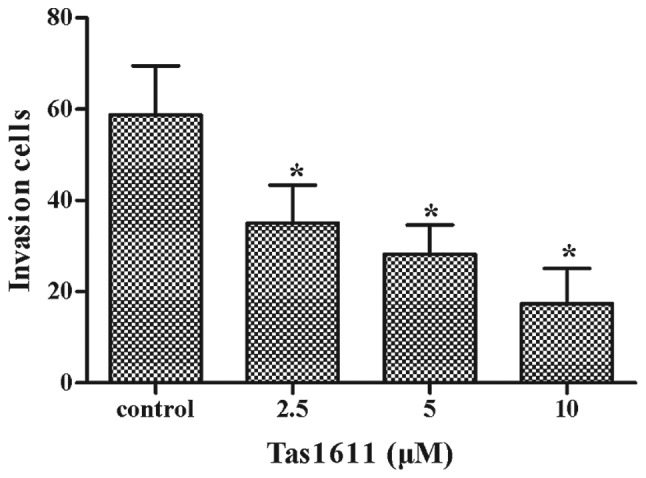
Quantization of the invasion assay. Data are presented as the mean ± SEM of three separate experiments. ^*^P<0.05 vs. control. SEM, standard error of the mean.

Cell invasion was assessed using a Transwell insert that contained polycarbonate membranes with 8-μm pores. The assay was performed using a Transwell that was precoated with Matrigel. The cells were treated with tas1611 or vehicle for 24 h, collected and allowed to migrate through the Matrigel-coated Transwell. As shown in Fig. 2, tas1611 inhibited the invasive abilities of the SMMC-7721 cells in a concentration-dependent manner. The mean cell invasion at 0, 2.5, 5 and 10 μM was 59±11, 35±8, 28±6 and 17±8, respectively.

### Tas1611 suppresses the activity of MMP-2 and MMP-9 in SMMC-7721 cells

**Figure 3:**
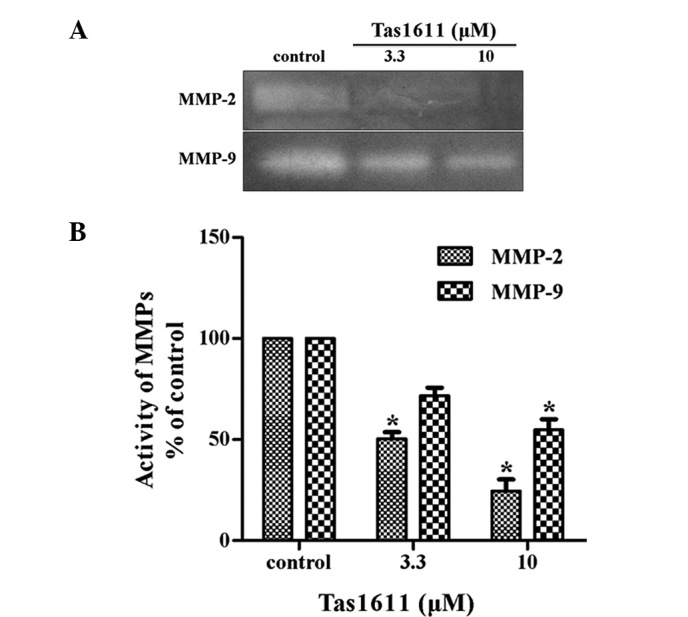
Effect of tas1611 on the activity of MMP-2 and MMP-9 in SMMC-7721 cells. (A) Gelatin zymogragpy analysis of serum-free media conditioned by the SMMC-7721 cells that were treated with tas1611. (B) Quantification of the gelatin zymography assay. Data are presented as the mean ± SEM of three separate experiments. ^*^P<0.05 vs. control. MMP, matrix metalloproteinase; SEM, standard error of the mean.

The potential effect of tas1611 pre-treatment on MMP-2 and MMP-9 secretion by the SMMC-7721 cells was examined using gelatin zymography. MMP-2 and MMP-9 activity in the SMMC-7721 cells was inhibited significantly by tas1611 pre-treatment (Fig. 3). The relative quantification of MMP-2 activity (percentage of control) was 50.40±3.29 and 24.51±5.77 at 3.3 and 10 μM, respectively. Similarly, MMP-9 activity was 71.74±3.98 and 54.86±5.17 at the same doses. This indicated that the inhibition of invasion by tas1611 was associated with the changes in gelatinase secretion or activation.

### Tas1611 downregulates the protein expression levels of MMP-2 and MMP-9 in SMMC-7721 cells

**Figure 4:**
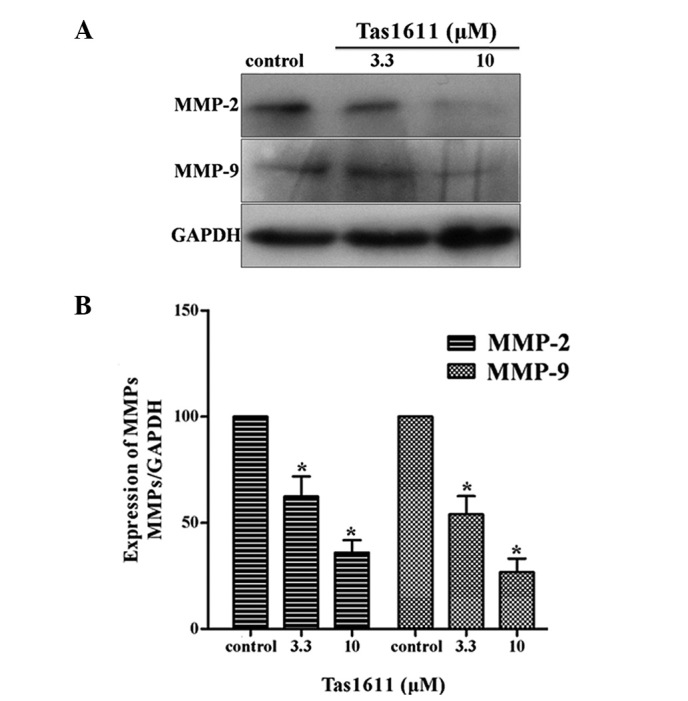
Effect of tas1611 on the expression of MMP-2 and MMP-9 in SMMC-7721 cells. (A) Western blot analysis of tas1611 in the downregulation of MMP-2 and MMP-9 expression in the SMMC-7721 cells. (B) Quantification of the western blot analysis. Data are presented as the mean ± SEM of three separate experiments. ^*^P<0.05, vs. control. MMP, matrix metalloproteinase; SEM, standard error of the mean.

Western blot anlaysis was performed to examine the protein expression of MMP-2 and MMP-9 in the SMMC-7721 cells, as shown in Fig. 4. The relative quantification of MMP-2 expression was 62.34±9.42 and 36.02±5.80 and the relative quantification of MMP-9 expression at the same dose of Tas1611 was 54.00±8.51 and 26.71±6.41, respectively. Tas1611 was observed to inhibit MMP-2 and MMP-9 protein expression in a dose-dependent manner compared with the control.

### Tas1611 downregulates the mRNA expression of MMP-2 and MMP-9 in SMMC-7721 cells

**Figure 5:**
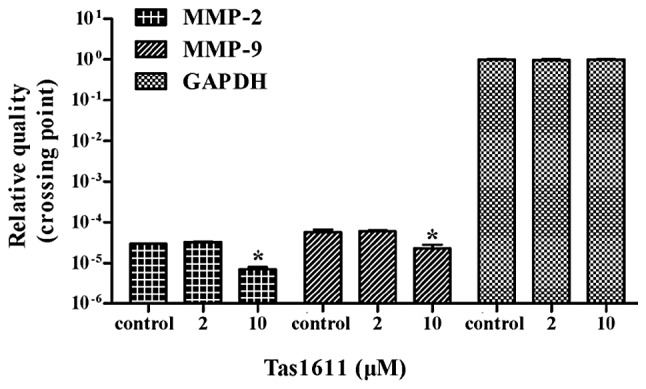
Effect of tas1611 on the expression of MMP-2 and MMP-9 mRNA in SMMC-7721 cells, as determined by quantitative PCR. The mRNA of MMP-2 and MMP-9 were inhibited in a dose-dependent manner compared with the untreated control. Values are expressed as mean ± SEM. ^*^P<0.05 vs. control. All the samples were run in triplicate. MMP, matrix metalloproteinase; SEM, standard error of the mean.

Quantitative PCR was performed to evaluate whether tas1611 was able to effect the synthesis of the MMP-2 and MMP-9 transcripts. The mRNA levels of MMP-2 and MMP-9 were both decreased by 66.6% at 10 μM (P<0.05). No significant difference was observed at 2 μM in MMP-2 or MMP-9 mRNA level (Fig. 5).

### Tas1611 inhibits tumor growth in an athymic mouse tumor model

The anti-tumor properties of tas1611 were evaluated using human tumor models that were xenografted in athymic mice. Tas1611 significantly inhibited tumor growth in the SMMC-7721 xenografted athymic mice in a dose-dependent manner and there was no substantial change in the body weight of the athymic mice during the experiment. Compared with the control group, the tas1611-treated group exhibited significantly inhibited tumor growth, with rates of 13.51 and 48.95%, respectively.

## Discussion

Invasion plays a critical role in tumor metastasis, which is the final stage of tumor progression (18). The evidence for MMPs, including MMP-2 and MMP-9, as active contributors to cancer progression arises from animal studies. Relatively benign cancer cells acquire malignant properties when MMP expression is upregulated. Conversely, highly malignant cells become less aggressive when MMP expression or activity is reduced (19).

The present study investigated the effect of the taspine derivative, tas1611, on the viability and invasion of SMMC-7721 liver cancer cells. The effect of tas1611 on the invasive properties of the SMMC-7721 liver cancer cells was investigated using a Matrigel chamber invasion assay. The results revealed that tas1611 demonstrated a marked inhibition of invasion in a concentration-dependent manner. The zymogram analysis of MMP activity showed that MMP-2 and MMP-9 activity was inhibited by tas1611 significantly. Tas1611 also downregulated the expression of MMP-2 and MMP-9 mRNA and protein levels. The results suggest that the anti-invasive action of tas1611 is partly mediated by diminishing the ability of cancer cells to degrade the components of the ECM by modulating the expression and activity of MMP-2 and MMP-9.

The *in vivo* effect on the growth of the SMMC-7721 cells that were xenografted in athymic mice was evaluated to test the efficacy of tas1611 on tumor inhibition. Compared with the control, the growth of the SMMC-7721 xenografts in the athymic mouse groups, which were treated with tas1611 at two different doses, were significantly inhibited. The final volume and weight of the xenografts were markedly reduced. This demonstrates that tas1611 plays a role in tumor inhibition.

Taken together, the results of the present study demonstrate that tas1611 was able to inhibit liver cancer cell growth and invasion. The compound was also able to reduce tumor growth in nude mice with xenografted SMMC-7721 cells. The mechanism underlying the invasion effect was attributed to the downregulation of MMP-2 and MMP-9 protein and mRNA levels. The data suggest that tas1611 is a potential candidate for an intervention against metastatic liver tumors, which otherwise lead to a higher mortality rate.
